# Esophageal obstruction secondary to enteral nutrition via nasogastric tube in moderate traumatic brain injury: A case report and literature review

**DOI:** 10.1097/MD.0000000000046755

**Published:** 2025-12-19

**Authors:** Qixin Jiang, Min Liu, Yu Liu, Peng Fu, Shilin Li, Lei Zha

**Affiliations:** a Intensive Care Unit, The People’s Hospital of Songtao Miao Autonomous County, Tongren, Guizhou Province, China; b Department of Graduate School of Bengbu Medical University, Bengbu Medical University, Bengbu, Anhui Province, China; c Department of Pulmonary and Critical Care Medicine, The First Affiliated Hospital of Wannan Medical College (Yijishan Hospital of Wannan Medical College), Wuhu, Anhui Province, China.

**Keywords:** 5% sodium bicarbonate solution, bezoar, enteral nutrition (EN), esophageal obstruction, traumatic brain injury (TBI)

## Abstract

**Rationale:**

Enteral nutrition via nasogastric (NG) tube is a standard intervention in intensive care units, particularly for patients with moderate traumatic brain injury (TBI). While effective, this approach may be associated with complications such as aspiration or feeding intolerance. However, esophageal bezoar formation remains an extremely rare complication.

**Patient concerns:**

We report a case involving a 50-year-old male with moderate TBI who underwent emergent craniotomy and decompressive craniectomy following intracranial hematoma. Postoperatively, the patient was managed in the intensive care unit with invasive mechanical ventilation and NG tube – administered enteral nutrition. On day 18 of enteral feeding, he developed acute esophageal obstruction.

**Diagnoses:**

Computed tomography and esophagogastroduodenoscopy confirmed a bezoar in the mid-to-upper esophagus composed of solidified enteral formula.

**Interventions:**

In vitro solubility testing identified 5% sodium bicarbonate as the most effective dissolution agent. Under endoscopic guidance, serial administration and aspiration of sodium bicarbonate successfully resolved the obstruction.

**Outcomes:**

Follow-up confirmed restoration of esophageal patency, and the patient recovered swallowing function prior to discharge.

**Lessons:**

This case underscores a rare but clinically significant complication of NG tube feeding in patients with moderate TBI. Prompt recognition, endoscopic confirmation, and tailored chemical dissolution therapy are essential for resolution. Preventive strategies, such as early consideration of post-pyloric feeding and careful monitoring of tube function, are warranted in high-risk populations.

## 1. Introduction

Nutrition therapy is a fundamental component of the comprehensive management strategy for critically ill patients, playing a pivotal role in improving clinical outcomes. Clinical data indicate that malnutrition is a common and serious concern in this population, and the early initiation of enteral nutrition (EN) has been shown to significantly enhance recovery and prognosis.^[1]^ Nasogastric (NG) tube administration remains a routine and widely accepted method of delivering EN in patients with intact upper gastrointestinal (GI) function, particularly those at nutritional risk in intensive care unit settings.^[2,3]^

Despite its widespread use and benefits, enteral feeding in critically ill patients is not without complications. Common adverse effects include diarrhea, aspiration, abdominal distension, and metabolic disturbances.^[4,5]^ Among these, the development of esophageal or gastric bezoars is a rare but clinically significant complication when EN is administered via an NG tube.^[6]^

A bezoar refers to an accumulation of undigested or partially digested material within the GI tract, most commonly the stomach, though it can also form in the esophagus or small intestine. Bezoars are categorized by composition into phytobezoars (plant material), trichobezoars (hair), pharmacobezoars (medications), and lactobezoars (milk products).^[7,8]^ Typical causes of esophageal obstruction include neoplasms, severe inflammation, congenital anomalies, and cysts. These generally present with progressive symptoms. Acute obstruction, in contrast, is often the result of foreign body ingestion or food impaction. In rare instances, retrograde migration of gastric contents into the esophagus may result in acute blockage.^[9]^

Although gastric bezoars are more frequently encountered, esophageal bezoars, especially those associated with EN, pose a higher risk for acute obstructive symptoms.^[10]^ Acute esophageal obstruction resulting from retrograde migration of gastric bezoars is exceedingly uncommon, with only isolated case reports available in the literature.^[11,12]^

This report presents a case of acute esophageal obstruction in a patient with moderate traumatic brain injury (TBI), secondary to solidification of enteral formula administered through a standard NG tube. The aim is to describe the diagnostic process, treatment strategy, and clinical outcome, and to provide a literature-based perspective on prevention and management of this rare complication.

## 2. Consent for publication

This case report was approved by the Ethics Committee of Songtao Miao Autonomous County People’s Hospital (approval number: 2024-10-13). Written informed consent for publication of the clinical details and imaging materials was obtained from the patient.

## 3. Case presentation

A 50-year-old male, referred to as patient Long XX, was admitted to the Neurosurgery Department on September 19, 2020, at 10:54 am, following a motor vehicle accident. He presented with complaints of headache, dizziness, and vomiting lasting approximately 2 hours. According to family members, the patient experienced a transient loss of consciousness immediately after the trauma, without evidence of seizures or urinary/fecal incontinence. He had no known history of chronic medical illness.

On admission, vital signs were as follows: blood pressure 187/125 mm Hg, heart rate 101 beats/min, respiratory rate 26 breaths/min, and temperature 37.0°C. Neurological examination revealed somnolence with a Glasgow Coma Scale score of 12 (E3V4M5). Pupils were equal in size (3 mm) and reactive to light. A non-contrast cranial computed tomography (CT) scan showed right cerebellar, bilateral frontal, and left temporoparietal cerebral contusions with associated hematomas, traumatic subarachnoid hemorrhage, a right occipital bone fracture, and bilateral old pubic rami fractures. The initial diagnoses included: moderate TBI, characterized by diffuse axonal injury, multifocal cerebral contusions, traumatic subarachnoid hemorrhage, occipital fracture, and scalp laceration; and aspiration pneumonia.

**Figure 1.**
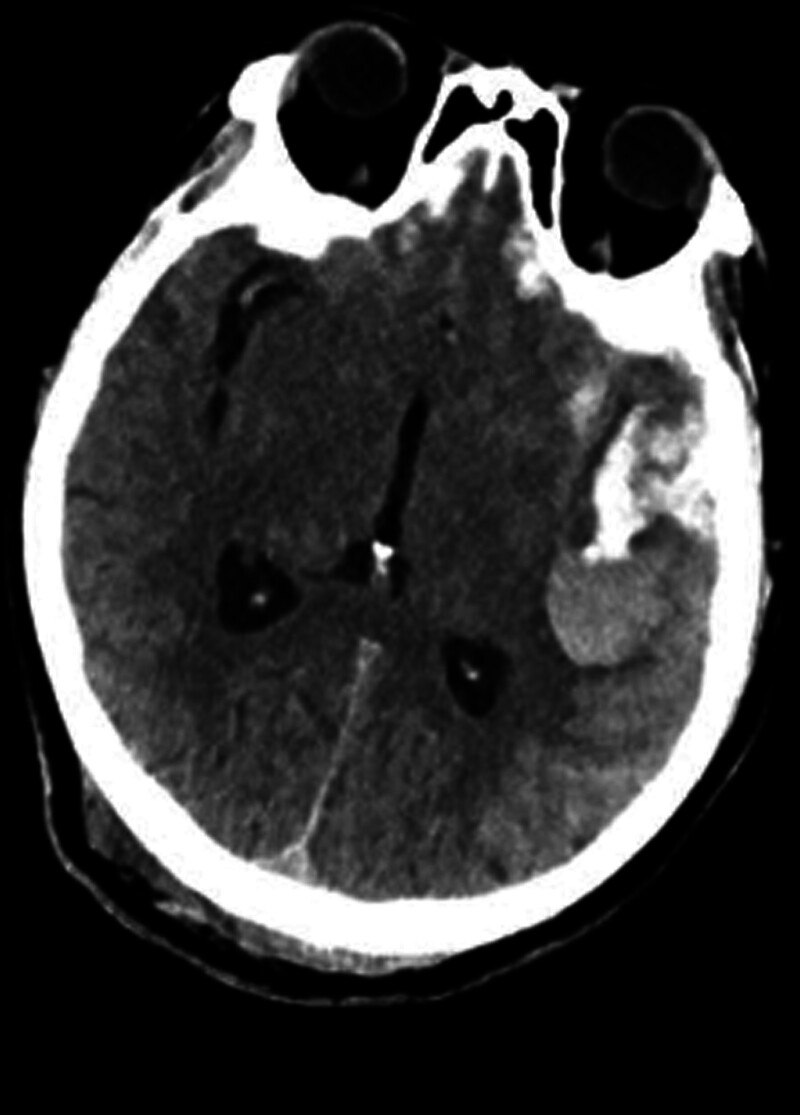
Head CT: compared with the morning head CT scan, the current hematoma has slightly increased in size and shows higher density. The subarachnoid hemorrhage remains unchanged. A pre-herniation state is suspected. CT = computed tomography.

**Figure 2.**
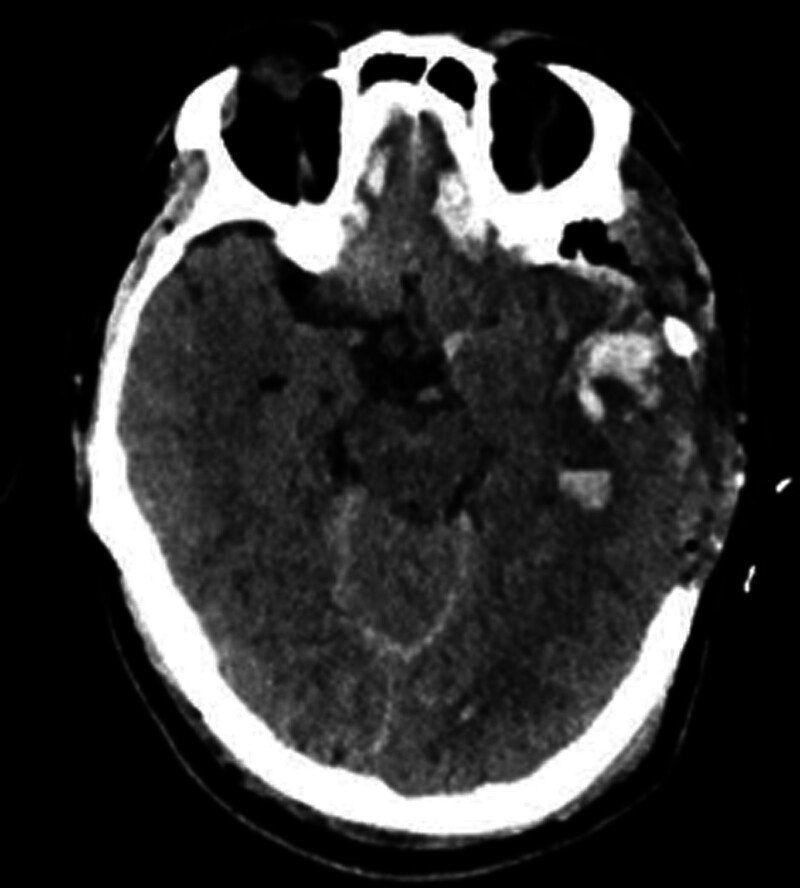
Head CT: no increase in intracranial hemorrhage. CT = computed tomography.

Following management with hemostasis, NG decompression, gastric protection, blood pressure control, analgesia, oxygen therapy, and vital sign monitoring, the patient’s level of consciousness deteriorated to a comatose state at 15:30 that day. An emergency head CT revealed enlargement of the hematoma with early signs of brain herniation (Fig. 1). The patient underwent urgent craniotomy with hematoma evacuation and decompressive craniectomy. Postoperatively, the patient was transferred to the intensive care unit for invasive mechanical ventilation. Follow-up head CT showed no significant abnormalities (Fig. 2).

On September 22, a tracheostomy was performed. EN (Fresubin^®^) was initiated via NG tube – which was used exclusively for EN without administration of any medications – at 20 to 30 mL/h, gradually titrated to 1000 mL/d based on tolerance, without early signs of feeding intolerance. He was successfully weaned from mechanical ventilation by September 27.

**Figure 3.**
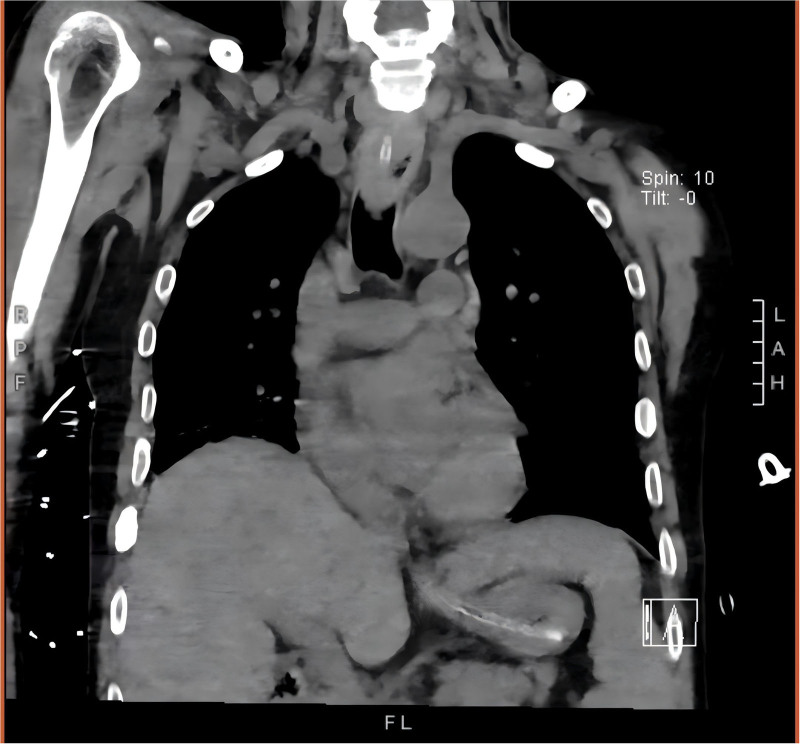
Abdominal CT: the tip of the nasogastric tube is positioned in the stomach. CT = computed tomography.

**Figure 4.**
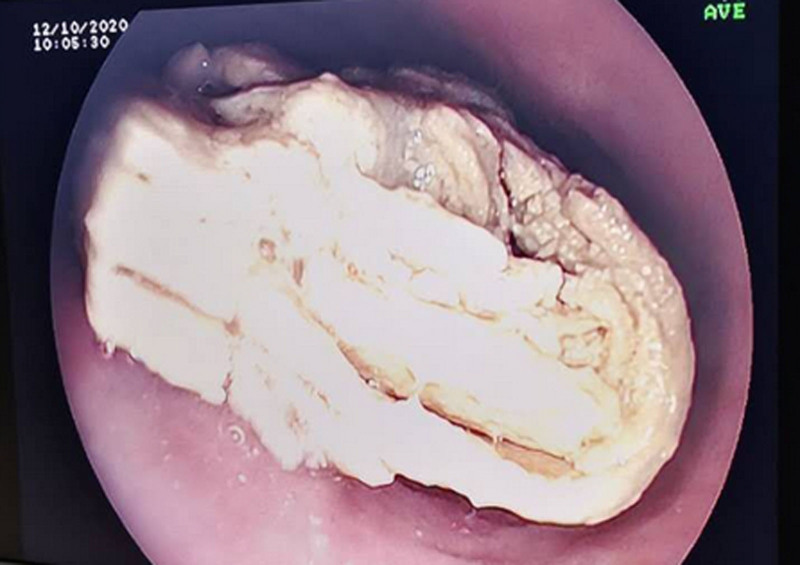
Gastroscopy: starting from 20 cm away from the incisors, a large amount of enteral nutrition residue was retained.

**Figure 5.**
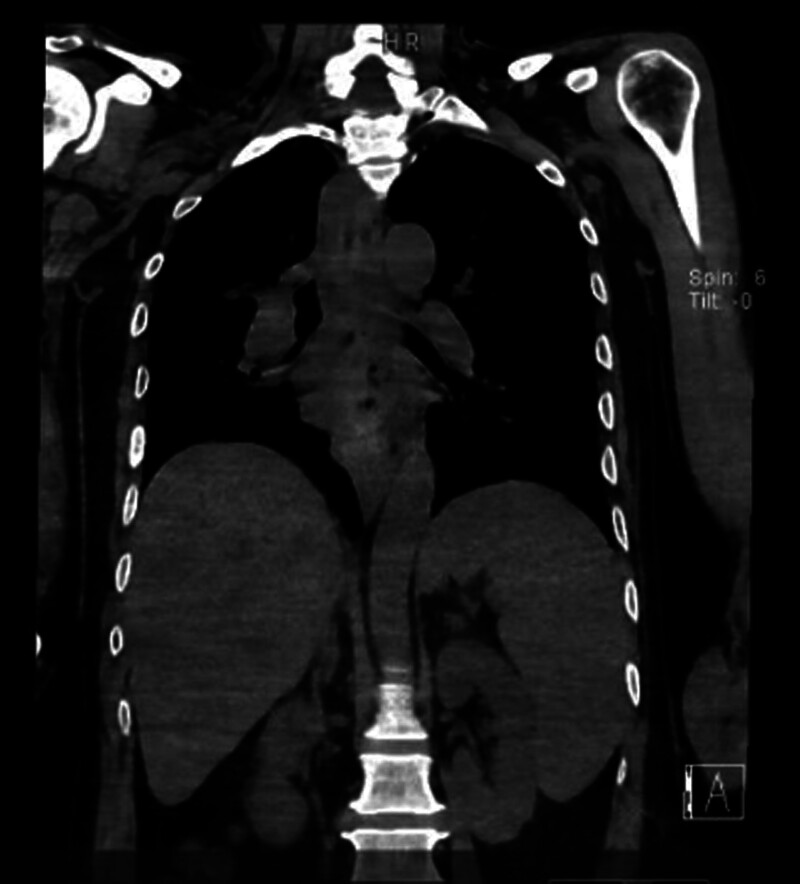
Three-dimensional CT: the esophagus revealed a large amount of retained contents in the middle and upper segments of the esophagus. CT = computed tomography.

An abdominal CT scan performed on October 3 demonstrated that the tip of the NG tube was located within the stomach (Fig. 3). However, on October 6, resistance was encountered during administration through the NG tube, which was found to be obstructed. Attempts at NG tube replacement were unsuccessful. Upper endoscopy on October 8 and a subsequent CT scan on October 10 identified a dense, obstructive mass of solidified enteral formula in the mid-to-upper esophagus (Figs. 4 and 5). Bedside esophagogastroduodenoscopy (EGD) confirmed a complete esophageal obstruction caused by bezoar formation.

**Figure 6.**
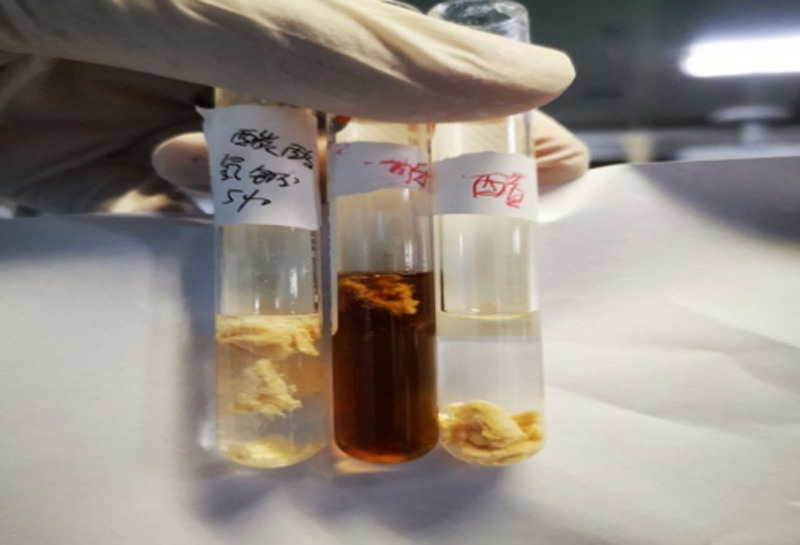
The obstructive substances could dissolve rapidly in a 5% sodium bicarbonate solution, but showed no significant change in vinegar and cola.

**Figure 7.**
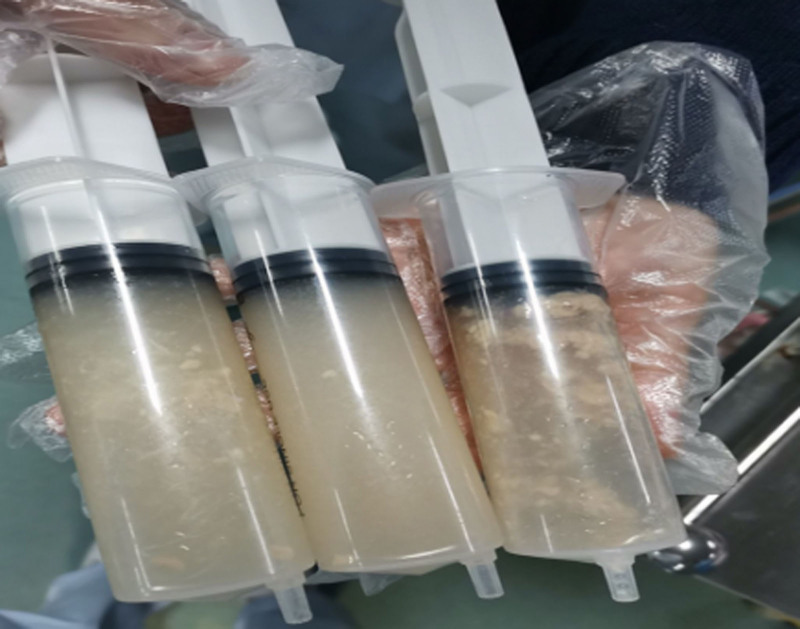
The procedure was repeated until the nasogastric tube smoothly entered the gastric cavity and thin nutrient solution was drained.

To identify an effective dissolution method, in vitro solubility testing was conducted using vinegar, cola, and 5% sodium bicarbonate solution. Only the 5% sodium bicarbonate demonstrated rapid dissolution of the impacted formula (Fig. 6). Under endoscopic guidance, repeated administration of 20 mL aliquots of sodium bicarbonate with concurrent aspiration successfully fragmented and cleared the bezoar (Fig. 7). Follow-up EGD on October 11 confirmed resolution of the obstruction. However, evidence of esophageal dysmotility prompted transition to post-pyloric feeding via nasojejunal (NJ) tube placement.

**Figure 8.**
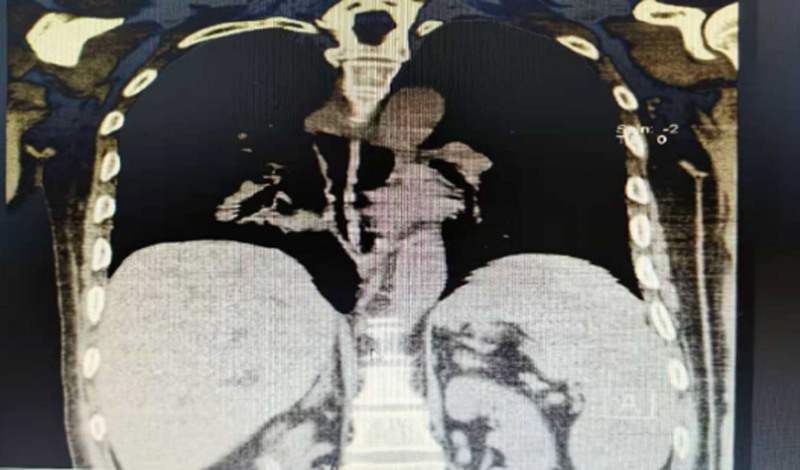
Coronal chest CT: follow-up chest CT scan demonstrated the disappearance of the foreign body in the esophagus, accompanied by significant esophageal dilation. CT = computed tomography.

**Figure 9.**
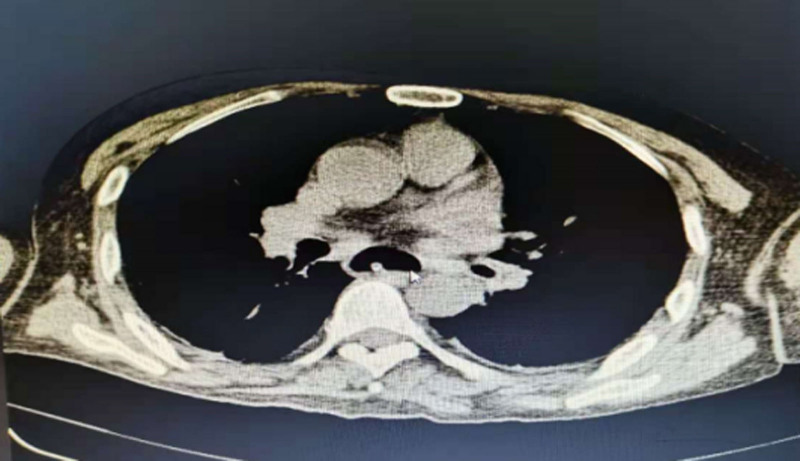
Axial chest CT: the axial view confirms the absence of the foreign body and clearly illustrates the circumferential esophageal dilation. CT = computed tomography.

Subsequent imaging confirmed resolution of the obstruction (Figs. 8 and 9). By November 2, the patient had regained adequate swallowing and chewing function and was able to resume oral intake. The NJ tube was removed, and he was discharged from the intensive care unit in stable condition.

## 4. Discussion

This case describes a rare instance of esophageal bezoar formation causing acute obstruction in a patient with moderate TBI, secondary to EN administered via a standard NG tube. Although EN is widely regarded as a safe, cost-effective, and physiologically appropriate method of nutritional support in critically ill patients, complications such as bezoar formation, particularly esophageal, remain exceedingly uncommon but clinically significant.^[13,14]^

Bezoars are categorized into 4 primary types based on their composition: phytobezoars (plant fibers), trichobezoars (hair), pharmacobezoars (medications), and lactobezoars (milk products).^[15]^ Esophageal bezoars may be classified as primary or secondary. Primary esophageal bezoars are generally associated with structural or functional abnormalities of the esophagus, such as achalasia, esophageal motility disorders, or neuromuscular diseases like myasthenia gravis and Guillain-Barré syndrome,^[16]^ and are more frequently observed in patients with compromised general condition or prolonged NG feeding.^[11,17,18]^

Secondary bezoars, in contrast, typically originate in the stomach and may migrate retrograde into the esophagus in the setting of severe vomiting, esophageal dysmotility, or chronic NG tube placement.^[9]^ In the present case, factors including prolonged EN administration, impaired esophageal motility (likely secondary to severe neurologic injury), and possibly altered gastric emptying contributed synergistically to bezoar formation and retrograde migration.

Prompt diagnosis of esophageal obstruction is critical, as delays may lead to aspiration, mucosal ischemia, or perforation. CT and EGD are the diagnostic modalities of choice. CT not only allows visualization of bezoar location and density but also helps confirm correct positioning of feeding tubes in critically ill patients.^[19]^ Urgent or emergent upper endoscopy is recommended based on the degree of obstruction, within 24 hours for partial obstruction and within 6 hours for complete obstruction.^[16,17]^ In this case, EGD played a pivotal role in confirming the diagnosis, guiding therapeutic intervention, and monitoring treatment response.

Treatment options for esophageal bezoars include both conservative and invasive approaches. Endoscopic fragmentation and removal remains the primary treatment. In patients unable to tolerate mechanical removal or at high procedural risk, chemical dissolution may be considered. Reported agents include carbonated beverages, acetylcysteine, enzymatic preparations such as cellulase or pancrelipase, and sodium bicarbonate.^[20,21]^ In this case, in vitro testing demonstrated that 5% sodium bicarbonate was superior to vinegar and cola in dissolving the enteral formula bezoar, leading to successful bedside endoscopic clearance.

This case underscores the importance of individualized monitoring of EN tolerance, especially in neurologically impaired patients who are at increased risk for GI dysmotility. Clinical parameters such as bowel sounds, gastric residual volumes, tube patency, and NG tube tip position should be regularly assessed. Preventive strategies include the elevation of the head of the bed to 30° to 45° during feeding, post-pyloric (NJ) feeding in high-risk patients, and careful attention to proper NG tube placement to avoid inadvertent esophageal feeding and reflux. Standardized protocols and staff training on the recognition and management of enteral feeding complications are also essential to reducing risk

## 5. Conclusion

Esophageal bezoar formation is an uncommon but clinically significant complication of NG enteral feeding in critically ill patients. Both primary and secondary mechanisms may lead to acute esophageal obstruction, particularly in patients with predisposing factors such as neurologic impairment, esophageal dysmotility, and prolonged NG tube use. A high index of clinical suspicion is warranted when resistance is encountered during medication or feeding administration through an NG tube.

Timely diagnostic evaluation, preferably with CT and EGD, is essential for identifying the cause, assessing the severity of obstruction, and guiding management. Endoscopic intervention remains the first-line therapeutic approach. In select cases, chemical dissolution using 5% sodium bicarbonate solution may offer a safe and effective nonsurgical alternative for bezoar clearance. Early recognition, appropriate intervention, and implementation of preventive strategies are critical to optimizing outcomes in this vulnerable patient population.

## Acknowledgments

We thank all the authors for their contributions in the study.

## Author contributions

**Conceptualization:** Qixin Jiang, Min Liu, Lei Zha.

**Data curation:** Qixin Jiang.

**Formal analysis:** Yu Liu.

**Funding acquisition:** Qixin Jiang, Lei Zha.

**Methodology:** Peng Fu.

**Resources:** Yu Liu, Peng Fu, Shilin Li.

**Validation:** Lei Zha.

**Writing – original draft:** Qixin Jiang.

**Writing – review & editing:** Qixin Jiang, Min Liu, Lei Zha.
